# ID 61 - Avaliações de Tecnologia em Saúde e Padronização de Material Técnico Hospitalar

**DOI:** 10.5327/2237-9622.2025.v34s1.61

**Published:** 2025-11-25

**Authors:** Rosa Amélia Durans Tavares, Valéria Regina Cavalcante dos Santos, Cinthia S. de Menezes da Silveira

## Abstract

**Introdução:**

O trabalho apresentado é um texto de divulgação científica para as redes sociais, e tem como principal iniciativa informar o papel das avaliações de tecnologias em saúde (ATS) no cenário hospitalar, que esta iniciando a implementação do Núcleo de Avaliação de Tecnologias em Saúde, assim como, de reforçar suas contribuições voltadas para a aquisição de novos padrões de materiais técnicos-hospitalares, fazendo um recorte da política e dos órgãos responsáveis, que norteiam o processo de avaliação.

A padronização de material técnico-hospitalar é um dos pontos de atenção da ATS, que visa contribuir com a redução do desperdício, a sustentabilidade dos recursos, a redução do risco de variabilidades, e a qualidade e a segurança do cuidado em saúde envolvendo pacientes e prestadores do serviço. A ATS no processo avaliativo de novas aquisições de materiais hospitalares fortalece a entrega positiva dos hospitais à sociedade, de um cuidado eficaz, resolutivo, seguro e de qualidade na transversalidade do cuidado em saúde.

**Método:**

Introdução; um breve histórico sobre ATS, a importância dos Nats no cenário hospitalar e a padronização de material técnico-hospitalar e o papel das ATS. no processo.

**Objetivos:**

Geral: Divulgar o papel das ATS no processo de padronização de material técnico-hospitalar. Específicos: Entender o processo dos Nats na avaliação de novas tecnologias; Apresentar a Política de Gestão de novas tecnologias voltadas para a saúde; Estabelecer a avaliação de padronização de materiais técnicos baseado nas orientações de ATS.

**Metodologia:**

Texto de Divulgação Científica nas redes sociais.

**Conclusão:**

Alcance e sensibilização das equipes envolvidas para a importância das ATS nos processos de aquisição de novas tecnologias em saúde.

**Equipamentos:**

Monitor e som para a apresentação do texto de divulgação científica.

